# Current progress on the microbial therapies for acute liver failure

**DOI:** 10.3389/fmicb.2024.1452663

**Published:** 2024-10-16

**Authors:** Jiayuan Huang, Tianyu Xu, Guoqiao Quan, Yuange Li, Xiaoya Yang, Wenrui Xie

**Affiliations:** ^1^Department of Gastroenterology, Research Center for Engineering Techniques of Microbiota-Targeted Therapies of Guangdong Province, The First Affiliated Hospital of Guangdong Pharmaceutical University, Guangzhou, China; ^2^Department of Physiology, Guangzhou Health Science College, Guangzhou, China

**Keywords:** acute liver failure, acute liver injury, type A hepatic encephalopathy, probiotics, microbiota, fecal microbiota transplantation, gut-liver-axis

## Abstract

Acute liver failure (ALF), associated with a clinical fatality rate exceeding 80%, is characterized by severe liver damage resulting from various factors in the absence of pre-existing liver disease. The role of microbiota in the progression of diverse liver diseases, including ALF, has been increasingly recognized, with the interactions between the microbiota and the host significantly influencing both disease onset and progression. Despite growing interest in the microbiological aspects of ALF, comprehensive reviews remain limited. This review critically examines the mechanisms and efficacy of microbiota-based treatments for ALF, focusing on their role in prevention, treatment, and prognosis over the past decade.

## 1 Introduction

Acute liver failure (ALF), characterized by prolonged prothrombin time and altered mental status, is a common condition involving rapid and severe damage to liver cells from various causes without cirrhosis. ALF results in liver failure, manifesting through coagulation abnormalities, altered mental status, peripheral vasodilation, and systemic inflammatory response, often culminating in multi-organ failure (Anand et al., 2020; Stravitz et al., 2023). ALF was initially termed “fulminant hepatic failure” in 1970; however, this term is now obsolete (Xue et al., 2022). Although the primary causes of ALF vary globally, the clinical presentations of patients are generally similar, attributed to the rapid onset of liver damage and the frequent complications such as infections, bleeding, and multi-organ failure within days. In cases of severe hepatic dysfunction, liver transplantation (LT) remains the definitive treatment for ALF, along with supportive therapy and care for associated complications (Kumar et al., 2021). However, fewer than 10% of patients with ALF receive LT due to factors such as shortage of donors, time constraints in patient evaluation before transplantation, and ethical concerns (Kumar et al., 2021). Consequently, the mortality rate of patients with ALF is high, reaching up to 80% in clinical practice (Zhao et al., 2013; Kumar et al., 2021). Thus, identifying effective alternative therapeutic interventions for ALF is essential.

The digestive tract harbors approximately 1,000 distinct bacterial species crucial for organismal survival. Marshall (1993) introduced the concept of the “gut–liver axis,” suggesting that a weakened intestinal barrier permits intestinal bacterial translocation and endotoxins to enter the portal vein system. Concurrently, inflammatory factors triggered by these harmful substances stimulate the activation of immune cells in the liver, producing and releasing inflammatory mediators that damage the intestinal mucosa and other organs. Moreover, growing evidence shows that the reciprocal relationship between the gut and liver plays a crucial role in various liver diseases (Jones and Neish, 2021).

Microbial therapeutics encompass a variety of approaches, ranging from non-targeted methods such as probiotics and fecal microbiota transplantation (FMT) to precision medicines targeting the microbiome, such as modified bacteria, postbiotics, and phages (Bajaj et al., 2022). The use of probiotic and microbial precision therapies for managing liver illnesses has advanced considerably (Bajaj et al., 2022). FMT is a therapeutic approach aimed at restoring the equilibrium of gut microbiota by transferring fecal microbiota from a healthy donor to a recipient. Since the first successful use of FMT by American scientist Eiseman in 1958 to treat four patients with severe pseudomembranous enterocolitis, its use in various medical conditions has steadily increased (Ooijevaar et al., 2019). A review by Prof. Gu concluded that FMT enhances recovery in chronic and acute liver diseases by efficiently altering intestinal microbiota composition (Gu et al., 2021).

As of March 2024, clinicaltrials.gov reported 150 registered trials on ‘Liver Diseases’ and ‘Microbiota,’ covering approximately 20 indications. Fatty liver disease, cirrhosis, liver failure, viral hepatitis, hepatic encephalopathy (HE), and autoimmune hepatitis represent the majority of the current research on the relationship between microbiota and liver disease. Articles on liver failure, most of which focused on acute-on-chronic liver failure (ACLF), have paid less attention to microorganisms in the context of ALF, with even fewer reviews addressing the subject. This review summarizes the potential of microbial therapies in ALF and examines the mechanisms underlying their use in the prevention, treatment, and prognosis of ALF over the past decade.

**Figure 1:**
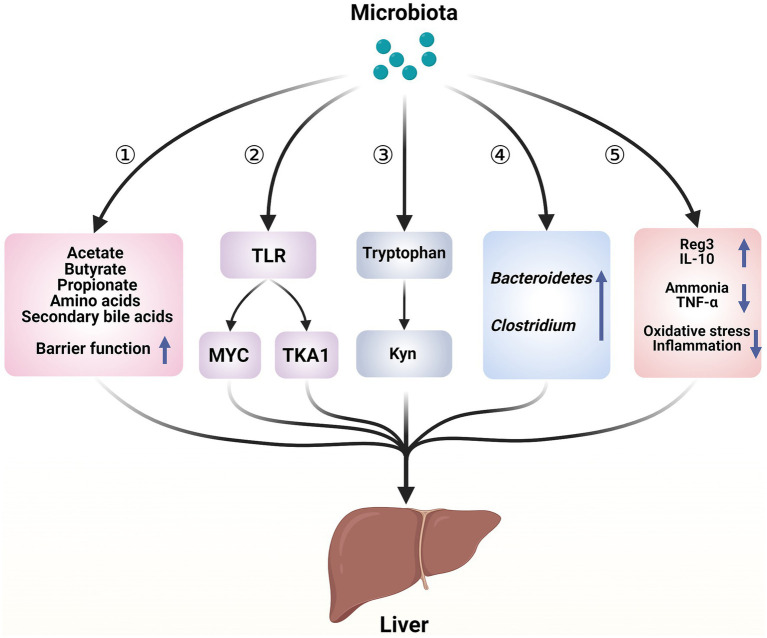
Hepatoprotective mechanisms of microbiota in acute liver illness. ① Microorganisms play a role in regulating liver function and contributing to the immune response in the liver by repairing the intestinal barrier and producing bioactive metabolites. ② Microorganisms cause the death of liver cells through a process mediated by TLR. ③ Microorganisms regulate the metabolism of tryptophan, which helps to adjust the damage to liver cells. ④ Enhancement of the bacterial community in the intestines. ⑤ Microorganisms release beneficial molecules, metabolize toxins, and reduce the inflammatory response and oxidative stress—created with BioRender.com.

## 2 Microbiome influence in the pathogenesis of acute liver disease

Probiotics and genetically modified bacteria influence liver function by altering the composition and behavior of gut bacteria. According to the gut–liver axis theory, the impact of gut bacteria on acute liver disease stems from the types of bacteria present and the toxic substances they produce (see Figure 1). In ALF, probiotics primarily function by increasing the abundance of potentially beneficial bacteria and reducing potentially harmful bacteria, thereby alleviating gut dysbiosis (Bloom et al., 2021).

Table 1 demonstrates that *Bacteroidetes* and *Clostridium* consistently exhibited higher abundance levels across several tests, indicating significant potential for further investigation. ALF occurs when liver cell death, whether through necrosis or apoptosis, exceeds the liver’s regenerative capacity (Rutherford and Chung, 2008).

**Table 1:** Application of microbiota in ALI or ALF models.

References	Probiotic	animal model	Protective mechanism	Gut microbiota
Wang et al. (2020)	*Bifidobacterium longum R0175*	Male Sprague–Dawley rats, ALF	Inflammatory response↓ Re-establishing of microbiota	Alloprevotella↑ Acetatifactor muris↓ Butyricimonas↓ Oscillibacter↓
Dong et al. (2022)	*Bifidobacterium longum BL-10*	Female BALB/c mice, ALI	Acetate↑ Butyrate↑ Inflammatory response↓ Intestinal injury↓ Hepatic oxidative damage↓ Re-establishing of microbiota	Clostridi↑ Lachnospiraceae↑ Lachnospiraceae↑ Desulfovibrio↓ Escherichia−Shigella↓
Li et al. (2019)	*Bifidobacterium adolescentis CGMCC 15058*	Male Sprague–Dawley rats, ALI	Inflammatory response↓ Intestinal barrier↑ Re-establishing of microbiota	Allobaculum↑ Bacteroidales↑ Coriobacteriaceae↑ Proteus↓
Fang et al. (2017)	*Bifidobacterium pseudocatenulatum LI09 and Bifidobacterium catenulatum LI10*	Male Sprague–Dawley rats, ALI	Inflammatory response↓ Intestinal injury↓ Bacterial translocation↓ Re-establishing of microbiota	Anaerostipes↑ Coprococus↑ Clostridium↑ Parasutterella↓
Jiang et al. (2021)	*Lactobacillus reuteri DSM 17938*	Male Sprague–Dawley rats, ALF	Inflammatory response↓ Retinol metabolism↓ PPAR signaling pathway↓ Intestinal tight junctions↑ Re-establishing of microbiota	Actinobacteria↑ Firmicutes↑ Actinomycetales↓ Coriobacteriaceae↓ Enterococcaceae↓ Staphylococcaceae↓
Cui et al. (2019)	*Lactobacillus reuteri ZJ617*	Male C57BL/6 mice, ALI	TLR4/MAPK/NF-κB activation↓ Apoptosis↓ Autophagy↓ Intestinal barrier↑	–
Yang et al. (2020)	*Lactobacillus salivarius Li01*	Male C57BL/6 mice, ALI	Inflammatory response↓ Intestinal tight junctions↑ Plasma and faecal ammonia levels↓ Neuro-inflammation↓ Re-establishing of microbiota	Akkermansia↑ Bacteroidetes↑ Proteobacteria↓ Ruminococcaceae↓
Zhuge et al. (2021)	*Lactobacillus salivarius Li01 + Bifidobacterium longum TC01*	Male Sprague–Dawley rats, ALF	Inflammatory response↓ Intestinal injury↓ Re-establishing of microbiota	Faecalibaculum↑ Eubacteria↑ Firmicutes/Bacteroidetes↓
Guo et al. (2023)	*Lactobacillus paracasei CCFM1222*	Male C57BL/6 mice, ALI	Inflammatory response↓ Oxidative stress↓ Cecal short-chain fatty acid (SCFAs) levels↑ Re-establishing of microbiota	Bifidobacterium↑ Faecalibaculum↑ Prevotellaceae↓
Wang et al. (2016)	*Lactobacillus casei Zhang*	Male Wistar rats, ALF	Inflammatory response↓ Myeloperoxidase activity↓ Intestinal injury↓ Re-establishing of microbiota	Bifidobacterium↑ Lactobacillus↑
Wang et al. (2019)	*Lactobacillus helveticus R0052*	Male Sprague–Dawley rats, ALF	Inflammatory response↓ Intestinal tight junctions↑ Re-establishing of microbiota	Bacteroides↑ Bacilli↑ Lactobacillales↑

Immunological pathways play a crucial role in the development of ALF regardless of etiology (Mann et al., 2023). During acute liver injury (ALI), intestinal bacteria utilize nutrients and metabolic substrates such as primary bile acids to produce various biologically active compounds, including acetate, propionate, butyrate, secondary bile acids, and amino acids. These compounds are absorbed into the liver via the portal vein and influence liver function through mechanisms involving secondary bile acids and microbial-associated molecular patterns (MAMPs) (Chen et al., 2021). Bile acids and MAMPs are integral to the development and maintenance of immunological responses in the liver (Chen et al., 2021). In an ALF rat model, reduced expression of RD-5, sPLA2, and lysozyme, along with decreased intestinal bacterial translocation in Paneth cells, suggests that ALF impairs the immunological barrier function of the ileum (Chen et al., 2020). Toll-like receptor (TLR) signaling has been identified as a link between bacteria and the epithelial barrier (Li et al., 2019).

Kolodziejczyk et al. (2020) conducted single-cell RNA sequencing on mice treated with thioacetamide or paracetamol, revealing the mechanism by which intestinal flora contributes to ALF. This study confirmed that detrimental intestinal bacteria contribute to the course of ALF by activating the TLR signaling pathway, thereby enhancing the activity of innate immune cells in the liver, such as MYC-dependent Kupffer cells. TLR4 signaling is the predominant factor in the progression of ALF. In animal models of ALF, TLR4 expression, which is responsible for detecting endotoxins produced by Gram-negative bacteria, is markedly elevated. Inhibition of TLR4 signaling has been demonstrated to mitigate organ damage and reduce systemic inflammation in ALF animal models (Yang et al., 2010; Engelmann et al., 2020). In addition, hepatotoxic substances, particularly lipopolysaccharides (LPS) transmitted through the portal blood, bind to TLR4, leading to either TGF-*β*-activated kinase 1 (TAKI) suppression or TLR4 activation. This induces extensive liver cell death during ALF through interactions with phagocytes via damage-associated molecular patterns (DAMPS) (Yang et al., 2010; Li W. et al., 2024).

Li D. et al. (2024); Li W. et al. (2024) and Zheng Z. et al., 2022 conducted a comprehensive metabolic analysis using ultra-performance liquid chromatography–mass spectrometry (UPLC-MS) and discovered that gut microbiota decreases tryptophan excretion and increases kynurenine (Kyn) release. Kyn, a naturally occurring compound, activates the aryl hydrocarbon (Ahr) receptor, reducing inflammation and inhibiting the immune response. Tryptophan, a compound found in the gut microbiota, plays a role in alleviating ALF in mice.

Table 1 demonstrates that the microbiota decreases inflammatory markers such as tumor necrosis factor-alpha (TNF-*α*) and interleukin-6 (IL-6) while increasing anti-inflammatory cytokines such as IL-10. TNF-α promotes programmed cell death of intestinal epithelial cells by activating caspase-3, which primarily contributes to increased permeability of the intestinal barrier in animals experiencing severe liver failure (Dewanjee et al., 2022; Xue et al., 2022; Zhou et al., 2022). Previous research has shown that the microbiota protects the liver from acute injury by reducing oxidative stress through various mechanisms. One such mechanism involves activating nuclear factor erythroid 2-related factor (2Nrf-2) signaling, stimulating the production of antioxidant enzymes. In addition, microbiota limits the production of reactive oxygen species (ROS) by generating metabolites that scavenge ROS and suppressing the activity of ROS-producing enzymes such as NADPH oxidase, CYP, and cyclooxygenase. Furthermore, microbiota enhance antioxidant metabolite levels in the host by binding metal ions (Wang Y. et al., 2017). They also inhibit microbial translocation by restoring the production of proteins that maintain tight connections between intestinal cells and preserve the structural integrity of the intestinal barrier. In summary, TLR4, IL-6, TNF-*α*, and Nrf-2 are crucial factors in every stage of ALF.

## 3 Microbiota prevent ALF

Previous research has demonstrated significant disruptions in the intestinal bacteria of animal models of ALF (Li et al., 2004). Patients undergoing long-term therapy with proton pump inhibitors or antibiotics are at a markedly higher risk of developing ALF due to dysbiosis (Schneider et al., 2021). Dysbiosis has been implicated in the pathogenesis of ALI or ALF following bariatric surgery (Moolenaar et al., 2022). Several agents, including selenium-enriched black garlic extract (Wu et al., 2024), Qingchangligan formula (QCLGF) (Yin et al., 2021), copper (Cu) (Chai et al., 2018), and oral magnesium (Li D. et al., 2024), have been shown to prevent ALF by indirectly modifying intestinal microbiota. Recent studies have demonstrated that various probiotics can effectively prevent ALF onset (Table 1). Reducing ALI and preventing ALF have emerged as a significant focus of microbiological research owing to the extremely high short-term mortality and rapid onset of ALF (Stravitz et al., 2023). *Bifidobacterium* and *Lactobacillus* are well-established probiotics that have been demonstrated to effectively prevent ALF in animal models (Chen, 2024).

### 3.1 *Bifidobacterium* in ALI

*Bifidobacterium*, the predominant gut flora, is crucial for maintaining a healthy microbial population. Previous studies highlight its beneficial properties, including its role in preventing intestinal infections (Hidalgo-Cantabrana et al., 2018), activating immune responses (Ménard et al., 2008), and participating in metabolic processes (Sugahara et al., 2015). Recent studies have shown that it has substantial potential to reduce liver damage (Jang et al., 2018; Kim et al., 2018). The primary species of *Bifidobacteria*, *Bifidobacterium longum*, has garnered significant scientific interest in ALF research (Wang et al., 2020; Zhuge et al., 2021; Dong et al., 2022). Previous studies demonstrate that *Bifidobacterium longum BL-10* alleviates oxidative liver damage, possesses 15 antioxidant genes with strong reducing abilities, and can efficiently eliminate 2,2-diphenyl-1-picrylhydrazyl (DPPH), hydroxyl radicals, and superoxide anions. Furthermore, bacterial metabolites such as acetic acid and butyric acid enhance anti-inflammatory factors and diminish pro-inflammatory factors by inhibiting the TLR4/NF-κB signaling pathway, reducing liver inflammation (Dong et al., 2022). Macrogene and correlation network analysis of rat fecal samples following pretreatment with *Bifidobacterium* elucidates the relationship between inflammatory cytokines associated with ALI and specific gut bacteria. The chemokine MIP-1α/MCP-1 negatively correlates with the *Odoribacter* genus in the treatment group that received *Bifidobacterium pseudocatenulatum LI09*, while the MIP-1α/M-CSF shows a negative correlation with *Flavonifractor* in the *Bifidobacterium catenulatum LI10* therapy group (Fang et al., 2017).

### 3.2 *Lactobacillus* in ALI

*Lactobacillus* enhances immune function, improving the body’s ability to combat infections, and activates specific immune cells in the liver and abdominal regions. This genus has been extensively studied in relation to liver disease (Osman et al., 2007; Jeong et al., 2022). *Lactobacillus reuteri,* a prevalent microorganism in the mammalian digestive system, is recognized as a probiotic (Widyarman et al., 2022). *Lactobacillus reuteri ZJ617* reduces hepatic TLR4/MAPK/NF-κB activation, decreasing apoptosis and autophagy in hepatocytes, thus preventing ALF (Cui et al., 2019). *Lactobacillus salivarius,* particularly the strain LI01, has also been investigated concerning ALF (Lv et al., 2014; Yang et al., 2020; Zhuge et al., 2021). *Lactobacillus plantarum* is closely associated with NF-κB regulation in ALF prevention, specifically through the MAPK/NF-κB or NLRP3/NF-κB signaling pathways (Duan et al., 2018; Li et al., 2021). Residual *Lactobacillus casei* and *Lactobacillus helveticus R0052* have also demonstrated the ability to inhibit ALF (Wang et al., 2019; Lv et al., 2024). However, not all *Lactobacillus* species confer liver protection. Notably, *Lactobacillus plantarum LI04* and *Lactobacillus salivarius LI02* do not significantly impact liver function, while *Lactobacillus paracasei LI03* worsens liver damage (Lv et al., 2014). These variations stem from differences in the sources and characteristics of the *Lactobacillus* species.

### 3.3 Other microbiota and potential side effects

In addition to *Bifidobacterium* and *Lactobacillus*, *Saccharomyces boulardii* also contributes to ALI mitigation by engaging in different clinical phases of the condition (Yu et al., 2017). A significant limitation in this research area is the lack of replication of specific probiotic regimens or model strains, reflecting the challenges faced in microbiological research across various fields (Bajaj et al., 2022; Koning et al., 2023). Therefore, the efficacy of specific microbiota in ALF needs to be further validated by many experiments.

Typically, the primary side effects of probiotics are gastrointestinal discomfort, such as diarrhea and bloating, arising from the inability of intestinal enzymes to break down oligosaccharides and polysaccharides, with osmotic characteristics being a primary cause (Davani-Davari et al., 2019; Hu et al., 2019). Most clinical trials and animal studies have not reported any severe adverse reactions associated with probiotics (Davani-Davari et al., 2019; Hu et al., 2019). However, individuals with significant immunological vulnerabilities, particularly older adults or newborns, face a higher risk of systemic infections such as bacteremia, sepsis, or endocarditis from probiotics (Davani-Davari et al., 2019). A case study noted that a newborn with an enlarged umbilical cord developed sepsis after 10 days of exposure to *Bifidobacterium breve* BBG01 (Ohishi et al., 2010). A double-blind, placebo-controlled clinical trial published in *JAMA* investigated the effects of a probiotic combination of *Lactobacillus rhamnosus GG* and *Bifidobacterium animalis* subsp. *lactis BB-12* in older nursing home residents (Butler et al., 2020). This study found that this probiotic combination did not significantly reduce the cumulative days of systemic antibiotic use. Furthermore, the probiotic group experienced a higher incidence of adverse events than the placebo group. These findings raise concerns, particularly when human factors or poorly designed experimental protocols are excluded. Thus, caution is warranted when using probiotics in specific populations.

## 4 Microbiota delay ALF

Currently, few studies have explored the use of microbial intervention in animal models or patients diagnosed with ALF. Type A hepatic encephalopathy (HE) is a severe complication of ALF, and its underlying causes are intricate and not yet completely understood. Although the traditional theory of ammonia toxicity is widely accepted, its exact mechanism remains unclear. Previous research has shown that hyperammonemia is effectively treated using microorganisms (Shen et al., 2015).

### 4.1 *Lactobacillus salivarius LI01* in ALF

*Lactobacillus salivarius LI01* prevents the development of ALF, delays its progression, and reduces the incidence of type A HE. Before inducing ALF in rats, oral administration of *Lactobacillus salivarius LI01* resulted in a delay in the course of ALF by modifying the composition of the intestinal microbiota, and there was a synergistic effect when *Lactobacillus salivarius LI01* was combined with *Bifidobacterium longum* (Zhuge et al., 2021). Prior administration of *Lactobacillus salivarius LI01* significantly reduced ammonia levels in both the blood and feces of rats. This intervention also improved cognitive function, reduced neuroinflammation, and positively influenced the expression of the brain-derived neurotrophic factor (BDNF) gene (Yang et al., 2020). Interestingly, varying doses of *Lactobacillus salivarius LI01* induced changes in the intestinal flora and metabolites, exhibiting a dose-dependent inverted U-shaped protective pattern (Lou et al., 2021). However, overuse of *Lactobacillus salivarius LI01* resulted in TAA-induced ALF mice or hyperammonemic mice being more susceptible to harmful gut bacteria. The optimal dosage and administration interval for *Lactobacillus salivarius LI01* remain unknown, warranting further research (Lou et al., 2021).

### 4.2 *Lactobacillus reuteri* in ALF

*Lactobacillus reuteri* prevents ALF, and certain strains also decrease the duration of ALF. The prophylactic oral administration of *Lactobacillus reuteri* improves the intestinal barrier in ALF rats by inhibiting the intestinal NF-kB pathway, which regulates the balance between Nrf-2 and NF-kB, thereby reducing intestinal inflammation (Zhou et al., 2022). Furthermore, the activation of Nrf-2/HO-1 via the PI3K/Akt and PKC pathways upregulates the anti-oxidative stress pathway and enhances anti-apoptosis in intestinal epithelial cells, further improving the intestinal barrier in ALF rats (Zhou et al., 2022). Prophylactic oral administration of *Lactobacillus reuteri R2LC* restores bacteria balance in the intestines by reducing bacteria translocation from the intestines to other parts of the body in mice with ALF, ultimately improving their condition (Wang et al., 1995). Before inducing ALF through modeling, another *Lactobacillus* strain, *Lactobacillus reuteri DSM 17938,* is orally administered to reduce liver damage through specific mechanisms, including lowering retinol metabolism and PPAR signaling pathways, interaction with viral proteins and cytokine receptors, and enhancement of central carbon metabolism in tumor signaling pathways (Jiang et al., 2021).

### 4.3 Other microbiota in ALF

Other *Lactobacillus* strains also slow the progression of ALF in mouse models. For example, oral administration of *Lactobacillus casei Zhang* serves as a preventive measure, slowing the course of ALF by influencing the TLR-MAPK-PPAR-*γ* signaling pathway, regulating the intestinal microbiota, and reducing liver inflammation (Wang et al., 2016). Previous research has also discovered that oral administration of *Bifidobacterium longum R0175* before inducing ALF decreases the release of several pro-inflammatory cytokines and maintains a healthy balance of gut bacteria (Wang et al., 2020). These results have a beneficial impact on ALF rat models.

### 4.4 Microbiota reduces high blood levels of ammonia

One of the most feared consequences of ALF is increased intracranial pressure and the resultant great effect, which may result from HE or cerebral edema, primarily caused by elevated ammonia (Paine and Pichler, 2023; Zhu et al., 2023). Lactulose remains the primary treatment for hyperammonemia. However, it is associated with significant adverse effects such as abdominal cramps, flatulence, bloating, and electrolyte imbalances (Liu et al., 2018). Alternative treatments such as rifaximin and sodium phenylacetate/phenyl butyric acid have shown limited effectiveness (Liu et al., 2018). Therefore, developing safer, more patient-friendly therapeutic strategies to lower blood ammonia levels is necessary. Modulating intestinal bacteria composition has become a standard practice for treating hyperammonemia and shows great potential in preventing the progression of ALF to HE (Liu et al., 2018). Ammonia is produced when the enzyme urease breaks down urea into carbon dioxide and ammonia (McCarville et al., 2020). Certain beneficial intestinal bacteria or probiotics inhibit urease activity or reduce its secretion, thereby decreasing ammonia production and restoring liver function (Zhu et al., 2023). In addition, emerging evidence suggests that microorganisms such as *Lactobacillus* repair the intestinal barrier and lower intestinal pH, thereby reducing ammonia absorption into the bloodstream (Bongaerts et al., 2005; Leal et al., 2023).

Previous research has identified the altered Schaedler flora (ASF), a group of eight bacterial species with the lowest number of urease genes. When antibiotics are used to deplete the native gut flora and replace it with ASF, urease activity takes several months to be restored, despite an increase in non-ASF species during this period (Mao et al., 2015; Shen et al., 2015). This approach establishes a long-lasting new community with reduced fecal urease activity and ammonia production.

Hong’s team (Liu J. et al., 2021) conducted a study using a radiotracer assay in a mouse model of hyperammonemia, investigating the ammonia removal process by probiotics in the intestinal lumen. They found that ammonia was removed by a probiotic strain called *Lactobacillus reuteri* JBD400; when combined with *Streptococcus rubneri JBD420*, this probiotic strain significantly improved transplantation efficiency and reduced ammonia levels. Another study discovered that an appropriate dosage of *Lactobacillus salivarius LI01* effectively treated acute hepatogenic hyperammonemia in a mouse model (Lou et al., 2021). In an experimental study using rodents with ALF, the wild-type strain NCIMB8826 of *Lactobacillus plantarum* was found to efficiently convert ammonia into alanine, aiding in regulating the absorption of ammonia in the gut and significantly improving survival rates (Nicaise et al., 2008). However, when the AmtB gene, responsible for transporting ammonia, is disrupted in *Lactobacillus plantarum*, its beneficial effect on reducing ammonia levels is significantly diminished (Nicaise et al., 2008).

Despite the potential benefits of microbial therapies in reducing blood ammonia levels, their efficacy and safety in critically ill patients with ALF remain inadequately studied. The ALF guidelines for 2023 highlight the lack of evidence supporting microbial therapies, including probiotics, for this patient population (Nanchal et al., 2023). Recent studies indicate that continuous renal replacement therapy effectively lowers serum ammonia levels and reduces mortality in critically ill patients with ALF and hyperammonemia (Warrillow et al., 2020; Paine and Pichler, 2023). However, further research is necessary to determine specific methods, intensity, and duration of treatment (Warrillow et al., 2020; Paine and Pichler, 2023).

### 4.5 Gel technology significantly enhances microbiological efficacy

Previous research has demonstrated that while various probiotics minimize liver injury, the harsh environment of the gastrointestinal system often limits their efficacy. This challenge has been effectively addressed by developing “gel technology” approaches (Zhuge et al., 2020). *Lactobacillus salivarius* LI01 encapsulated in alginate-pectin (AP) microgels significantly enhances the hepatoprotective activity of probiotic colonies in simulated gastrointestinal fluids and bile salt solutions during long-term storage and heat treatment (Zhuge et al., 2020). Another hydrogel particle, shaped like a seed and coated with lignin, is designed to retain and protect against bacteria while facilitating diffusion and improving intestinal microbial adaptation (Zheng D. et al., 2022). Gel particles have successfully boosted the efficacy of microbes in both mouse and porcine models of ALF (Zheng D. et al., 2022).

In summary, certain microorganisms can both prevent and delay ALF. Modifying the balance between Nrf-2 and NF-kB is a crucial mechanism through which these microorganisms exert their effects. Previous research has primarily focused on the timing of microbiota intervention during the pre-modeling phase of ALF in animal models. While it has been shown that ALF-affected mice exhibit disrupted intestinal flora (Li et al., 2004), studies specifically investigating microbiome therapies in the early stages of ALF in animal models or humans are lacking. Therefore, further investigation is warranted.

## 5 Microbiota enhances the prognosis of ALF

Multiple studies have shown that microbial treatment significantly improves survival rates in patients with ACLF without requiring LT (Abbas et al., 2022). In addition, it slows disease progression, reducing the need for LT (Abbas et al., 2022). Recent research has highlighted that microorganisms are crucial in delaying disease progression and preventing HE in individuals with ALF. While LT remains the only proven treatment to enhance survival in patients with ALF, the prognosis of LT in patients with ALF remains challenging, with survival rates typically lower than those seen in patients undergoing elective LT for chronic liver conditions (Kumar et al., 2021; Stravitz et al., 2023). A 2023 article in the *American Journal of Transplantation* outlined the primary mechanisms by which microorganisms influence LT outcomes (Sucu et al., 2023).

### 5.1 Microbiota decreases infections associated with LT

Infections following LT are common complications, with rates ranging from 30 to 86%, and contribute to higher mortality rates and extended hospital stays (Rayes et al., 2005; Sawas et al., 2015). Bacterial translocation is a crucial factor in post-LT infections (Mu et al., 2019).

Administering microbiological agents, such as probiotics or prebiotics, before LT effectively reduces postoperative infections, especially when administered shorter after surgery (Rayes et al., 2005; Sawas et al., 2015). In addition, the combination of fiber and *Lactobacillus* further reduces the incidence of bacterial infections after LT, shortens the duration of antibiotic therapy, and prevents antibiotic resistance when compared to conventional nutritional support (Zhang et al., 2013). Synbiotics containing multiple *Lactobacillus* strains and fiber were found to be more effective in preventing infections than synbiotics containing single-strain *Lactobacillus* and fiber due to their synergistic effects (Rayes et al., 2005). An 18-month randomized, double-blind, placebo-controlled trial demonstrated that Prowel, a novel synbiotic developed by Shweta Mallick’s team, effectively reduced complications, including infections, during the initial postoperative phase after LT (Mallick et al., 2022). Early enteral nutrition (EN) and fiber formulations with specific prebiotics (e.g., *Lactobacillus plantarum*) have also been recommended in the newly released ESPEN practical guidelines to lower post-LT infection rates (Weimann et al., 2021).

### 5.2 Microbiota improves nutritional status

Emerging evidence suggests that disturbance of microbiota growth is a significant causal element in malnutrition (Gehrig et al., 2019). A review published in *Science* proposed that immature gut microbiota causes pathogen invasion, exacerbating dysbiosis and ultimately affecting bioregulatory systems, including muscle and bone growth. However, the underlying mechanisms remain unclear (Blanton et al., 2016). Storelli et al. discovered that *Lactobacillus plantarum* stimulated development in a drosophila model of diet-induced stunting by regulating hormonal signaling through TOR-dependent nutrient sensing (Storelli et al., 2011). In 2023, a study conducted by the team further confirmed that *Lactiplantibacillus plantarum* LpWJL stimulated the growth of small intestinal crypt cells by promoting cell proliferation and type 1 interferon signaling through interactions with the pattern-recognition receptor NOD2 in the intestinal epithelium (Schwarzer et al., 2023). Similar growth-promoting effects were observed with inactivated *Lactiplantibacillus plantarum* LpWJL and its cell wall extracts, such as cytosolic acyl dipeptides and mifamurtide (Schwarzer et al., 2023). Recent interest in microbiome-based personalized nutritional regimens reflects the growing understanding that microbes play significant roles in the nutritional status of patients (Kolodziejczyk et al., 2019; Vandeputte, 2020).

Although nutritional deficits and metabolic issues improve after LT, patients’ nutritional status can rapidly decline due to surgical stress, perioperative malnutrition, intense postoperative protein breakdown, and fasting (Merli et al., 2010; Weimann et al., 2021). Thus, the use of microbial agents, such as probiotics and prebiotics, in nutritional therapy for patients undergoing LT is beneficial for addressing malnutrition and muscle loss and also for improving psychiatric symptoms (Anastácio and Davisson Correia, 2016).

### 5.3 Microbiota affects hepatocyte regeneration

Hepatocyte regeneration often follows liver injury, although many liver disorders inhibit this process (Forbes and Newsome, 2016). Hepatic resection leads to substantial alterations in over 6,000 bacterial genes, such as an increase in *Bacteroidetes S24-7* and *Rikenellaceae* and a decrease in *Firmicutes, Clostridiales*, *Lachnospiraceae*, and *Ruminococcaceae*, all of which are strongly linked to hepatocyte regeneration (Liu et al., 2016a). Recent reviews have shown that gut microbiota signaling through the portal vein plays a role in hepatocyte regeneration (Zheng and Wang, 2021; Xu et al., 2022). Metabolites produced by the gut microbiota, such as bile acids, short-chain fatty acids (SCFAs), tryptophan derivatives, and probiotics, impact host metabolism and immune function, indirectly affecting hepatocytes (Garrett, 2020; Van Treuren and Dodd, 2020; Cohen et al., 2023). Notably, during the initial stages following severe liver injury or post-hepatectomy, excessive bile acid absorption from the intestines can hinder hepatocyte repair and may induce secondary liver injury (Zheng and Wang, 2021).

While inflammatory mediators such as IL-6 and TNF-*α* promote inflammation, they also promote hepatocyte regeneration. IL-6 binds to glycoprotein 130, a second receptor protein, and activates the JAK/STAT pathway, promoting early genes linked to mitosis and regeneration, allowing quiescent hepatocytes to transition from G0 to G1 and S phases (Hibi et al., 1990; Heinrich et al., 2003; Schmidt-Arras and Rose-John, 2016; Naseem et al., 2018). In addition to activating the TNF receptor on the surface of Kupffer cells via autocrine action to upregulate NF-kB to activate IL-6 transcription, TNF-*α* also promotes the expression of cell cycle protein D1, a key cell cycle promoter in hepatocytes via MAPK–ERK and c-Jun N-terminal kinase (JNK) (Taki-Eldin et al., 2012). LT-induced ischemia alters the intestinal microbiota, regulated by immune mediators such as IL-6, leading to inflammation and immune responses that ultimately affect hepatocyte regeneration. Commercial probiotics promote postoperative hepatic tissue repair after liver surgery by reversing these pathways (Cornide-Petronio et al., 2020; Cohen et al., 2023). Although LPS, a crucial component of Gram-negative bacteria, is toxic to the liver (Shi et al., 2022), moderate release of LPS by the intestinal flora has been shown to promote hepatocyte regeneration by triggering the conversion of C3 and C5 into bioactive peptides called C3a and C5a, which interact with Kupffer cell receptors to stimulate the release of TNF-α (Marshall et al., 2014).

Preoperative nutritional state before LT, including cholesterol levels, impacts ischemia–reperfusion (I/R) outcomes, postoperative metabolism, and liver regeneration because retinoic acid plays a role in regulating the *Firmicutes-to-Bacteroidetes ratio* (*F/B*) and modulating the FGF21-LKB1-AMPK pathway, which promotes energy metabolism and facilitates liver regeneration (Saïdi et al., 2015; Silva et al., 2015; Liu et al., 2016b). Peng et al. reached a similar conclusion, successfully regulating the gut microbiota by feeding *Lactobacillus plantarum* AR113 to rats for 2–3 weeks. This intervention reduced the F/B ratio, which subsequently affected plasma glycerophospholipids and ultimately accelerated liver regeneration in a hepatectomized rat model (Xie et al., 2021).

## 6 The use of FMT and engineered bacteria in the field of ALF

Ongoing advancements in microbe-based therapies that positively influence all aspects of ALF include engineered bacteria and FMT, which are among the most rapidly developing emergent technologies.

### 6.1 FMT

Although the effectiveness and safety of FMT have been demonstrated for treating cirrhosis, chronic liver disease, and ACLF, there is limited research on its use for ALF (Trebicka et al., 2021; Won et al., 2022). A randomized phase I clinical trial involving 10 patients with cirrhosis showed that FMT administered via enema restored gut microbiota diversity and improved hyperammonemia. However, it is essential to note that the patients also received broad-spectrum antibiotics in conjunction with FMT, which limits the applicability of the findings (Bajaj et al., 2017). In addition, a phase I pilot study of oral FMT capsules reported that FMT also restored microbial diversity in the duodenal mucosa and intestines of patients with HE. This restoration was associated with decreased LPS-binding protein (LBP) levels and increased expression of antimicrobial peptides (Bajaj et al., 2019).

The potential mechanism through which FMT reduces blood ammonia levels was highlighted in a 2023 preprint, describing how FMT reduces microbial-associated ammonia production by enhancing the anaerobic metabolism of L-aspartic acid and increasing ammonia utilization. This was observed independent of concurrent rifaximin therapy or antibiotic pretreatment (Shawcross et al., 2023).

Based on this theoretical basis, Wan et al. demonstrated that transplantation of healthy human feces via FMT into a rat model of CCL4-induced liver failure reduced inflammatory response mediators such as TLR4 and TLR9 and circulating pro-inflammatory factors such as IL-1β, IL-6, and TNF-α It also helped restore tight junction proteins in the intestinal mucosal barrier, reduced blood ammonia levels, ameliorated liver necrosis, and improved behavior and spatial learning ability in the animal model, ultimately preventing the occurrence of HE (Wang W. W. et al., 2017). However, the simulated experiment lasted 9 weeks, exceeding the ALF threshold of 2 weeks (You et al., 2013), technically modeling subacute liver failure.

Another study validated the role of FMT in restoring intestinal flora balance and regulating the interplay between pro-inflammatory and anti-inflammatory cytokines, including Th17/Treg cytokines, thereby moderating ALI induced by d-GalN in mice (Liu Y. et al., 2021). In 2023, Yuan et al. (Yuan et al., 2023) discovered that transferring healthy human feces into a mouse model with ALF through FMT effectively decreased the level of caspase-3 cleavage, decreasing hepatocyte apoptosis caused by toxins. In addition, correlations between the composition of the microbiota and hepatic metabolites were observed using Pearson’s correlation coefficients.

Despite the benefits, traditional FMT delivery through colonoscopy and the resulting patient discomfort has hindered its advancement in the domain of ALF treatment. In 2023, the FDA introduced Vowst (SER-109), the first oral FMT, designed primarily to prevent recurrent *Clostridium difficile* infections (CDIs) in adults (Blair, 2024). An investigation published in 2024 provided evidence that an innovative oral-fecal gavage (OFG) preserves microbial activity, thereby contributing to the relief of ALI in a murine model (Yang et al., 2024). In contrast to conventional FMT, OFG offers enhanced convenience, gentler administration, and greater acceptability, making it more suitable for delaying the progression of ALF in the future.

### 6.2 Engineered bacteria

Researchers have recently focused on creating more intelligent engineered bacteria by modifying probiotics. These modified bacteria are capable of detecting and reacting to signals in their surrounding microenvironment to achieve more accurate and efficient treatments (Zuo and Zhao, 2023).

Engineered bacteria produce molecules that positively affect the host, such as increasing antimicrobial activity in the gut through the secretion of Reg3 and exerting immunomodulatory properties on immune cells in the lamina propria through the secretion of IL-10, as well as metabolizing harmful substances such as ammonia, converting it into L-arginine (Steidler et al., 2000; Hendrikx et al., 2019; Kurtz et al., 2019; Bajaj et al., 2022; Zuo and Zhao, 2023). Genetically modified microorganisms have shown significant hepatoprotective properties. In a mouse model of liver disease caused by ethanol, the introduction of a modified version of *Lactobacillus reuteri,* which produces the anti-inflammatory protein IL-22, resulted in increased levels of antimicrobial substances, such as Reg3g, in the intestinal epithelial cells. This prevented bacteria translocation from the intestine to the liver and reduced the damage caused by ethanol (Hendrikx et al., 2019). A study conducted in 2023 (Kouno et al., 2023) revealed a specific mechanism involving IL-22. By introducing a newly developed bacterium into a mouse model of ethanol-induced liver disease, it produced tryptophan metabolites, namely, 3-indoleacetic acid (IAA) and 3-indole-lactic acid (ILA), which bind Ahr immune cells in the lamina propria. This increased the production of IL-22, stimulating the production of the antimicrobial substances Reg3g and Reg3b in the intestinal epithelium, thereby decreasing the number of bacteria in the mucus layer and preventing bacterial translocation, ultimately protecting the liver. Hence, engineered bacteria associated with the tryptophan metabolite/IL-22/Reg3g metabolic pathway play a distinct role in hepatoprotection and may prove advantageous for patients with ALF. In addition, a synthetic bacterium called EcN-MT alleviates hepatic damage in mice exposed to cadmium by utilizing expressed metallothionein to bind cadmium and substantially increase the population of beneficial bacteria, including *Lactobacillaceae* (Zou et al., 2022).

Engineered bacteria also effectively mitigate HE by reducing blood ammonia levels. Genetic engineering has been employed to construct a high ammonia-consuming *Lactobacillus plantarum* strain by artificially inducing inactivation of lactate dehydrogenase (LDH) and knock-in of alanine dehydrogenase (AlaD) genes. This strain is more effective in lowering hyperammonemia in an animal model of ALF than the wild-type strain (Nicaise et al., 2008). Synlogic introduced an engineered bacterium strain SYNB1020 from Probiotic *Escherichia coli (E. coli)* Nissle 1917. This strain mitigates hyperammonemia and enhances survival in a mouse model of thioacetamide-induced hepatic injury with hyperammonemia by enhancing the L-arginine synthesis pathway. Experiments on healthy populations observed that SYNB1020 demonstrates active metabolism and facilitates the urea cycle, clarifying its pharmacokinetic mechanisms (Kurtz et al., 2019). Regrettably, despite the successful completion of a phase I clinical trial involving this strain (NCT03179878), SYNB1020 could not complete a subsequent phase Ib/IIa trial (NCT03447730), which was terminated due to reported inefficacy (Rutter et al., 2022). Fortunately, SYNB1536, a more viable option for preventing HE, is an innovative engineered bacterium derived from *E. coli.* This strain augments the pathway for L-arginine synthesis and introduces butyric acid synthesis, contrasting with SYNB1020. In addition to its ammonia-lowering capability, this bacterium exhibits anti-inflammatory, antioxidant, and intestinal barrier protection and safeguards brain function due to the secretion of butyric acid (Ochoa-Sanchez et al., 2021).

In summary, both FMT and modified microbes show significant potential for use in ALF. Although preclinical models have demonstrated these advantages, the efficacy of most of these microorganisms has not yet been substantiated in clinical studies. Therefore, they should undergo additional evaluations in patients with ALF, hyperammonemia, or HE.

## 7 Conclusion and outlook

Substantial evidence supports using microorganisms to treat the various stages of ALF. *Bifidobacterium* and *Lactobacillus*, particularly *Lactobacillus salivarius LI01*, show considerable potential for successful clinical outcomes. However, specific dosages and administration timing need further investigation. Other bacteria, such as *Bacteroidetes*, *Clostridium*, and engineered bacteria, also hold significant potential for ALF treatment.

Due to the rapid onset and high mortality rate of ALF, moderate OFG offers a better treatment option compared to traditional FMT. While most ALF and microbiological investigations focus on animal models, predominately involving male subjects, further clinical trials are needed to determine the applicability of these findings to humans. As humanized animal models become more accessible, the gap between animal and human disease models has diminished (Walsh et al., 2017; Guo et al., 2018). There is less application in liver disease because indications for microbial clinical studies are mainly focused on diarrhea, constipation, and dyspepsia in the digestive system. The major indications for microbiological clinical studies in liver disease include metabolic dysfunction-related fatty liver (MASH), non-alcoholic fatty liver (NAFLD), and liver cancer. However, only VSL#3 is currently approved for sale, and Research and Development (R&D) phase for other products is still in the clinical research stage (Table 2).

**Table 2:** Application of microbiota in clinical trials of liver diseases.

Name	Country	Company	Indication about liver	Drug type	Protective mechanism	The highest R&D phase in the world is underway (Clinical trial number)	The highest R&D phase for liver diseases in the world is underway (Clinical trial number)
Live lactic acid bacteria probiotics (other names:VSL-3, VSL#3) (Zhuge et al., 2021)	America	Original/Active company: Vsl Pharmaceuticals Inc	NAFLD; HE; Cirrhosis	Multiplex probiotic	Consisting of 8 different probiotic strains, including *Lactobacillus* and *Bifidobacterium*, to regulate intestinal microbiome	NAFLD:Marketing approval	NAFLD:Marketing approval HE: clinical stage 1 (ChiCTR2000040960) Cirrhosis: clinical stage 2 (EUCTR2010-022886-92-GB; NCT01701297)
VE303 (Louie et al., 2023)	America; Canada	Original/Active company: Vedanta Biosciences Inc	HE	*Viable* microbial	Consortium of 8 strains of commensal *Clostridia* to regulate intestinal microbiome	Clostridioides Difficile Infection: clinical stage 3 (NCT06237452)	HE: clinical stage 2 (NCT04899115)
*Faecalibacterium prausnitzii* EXL01	Poland; Belgium; France	Original company: Exeliom Biosciences	Liver cancer	Microbial extracts	IL10 stimulator; Regulating intestinal microbiome	Liver cancer: clinical stage 2 (NCT06551272)	
		Active company: Center Eugene Marquis; Exeliom Biosciences; Gercor—Multidisciplinary Oncology Cooperative Group					
Oral enterobacterium capsules	China	Original/Active company: Nanjing Xieshou Biotechnology Co. Ltd	Liver cancer	Microbial agent	Regulating intestinal microbiome	Liver cancer: clinical stage 2 (NCT06563934, NCT06563947)	
RBX7455	Switzerland; America	Original company: Rebiotix, Inc	HE; Cirrhosis	Microbiota capsule	Regulating intestinal microbiome	Cirrhosis associated HE: clinical stage 2 (NCT04155099)	
		Active company: Ferring BV					
PRIM-DJ2727	America	Original/Active company: University of Texas Health Science Center at Houston	NAFLD	Microbiota capsule	Regulating intestinal microbiome	NAFLD: clinical stage 2 (NCT04371653)	
Fecal microbiota	Germany	Original/Active company: Universitätsklinikum Heidelberg	Refractory liver cancer	FMT	Regulating intestinal microbiome	Liver cancer: clinical stage 2 (NCT05690048)	
Fecal microbiota	Austria	Original/Active company: Medical University of Vienna	Refractory liver cancer	FMT	Regulating intestinal microbiome	Liver cancer: clinical stage 2 (NCT05750030)	
Fecal microbiota	America	Original/Active company: Lifespan Corp	MASH	FMT	Regulating intestinal microbiome	MASH: clinical stage 1 (NCT02469272)	
MH008	Belgium	Original/Active company: MRM Health NV	MASH	*Viable* microbial	Regulating intestinal microbiome	*Preclinical application*	
MNO-163; MNO-863	China	Original/Active company: Moon (Guangzhou) Biotech Co. Ltd	MASH	*Viable* microbial	Regulating intestinal microbiome	*Preclinical application*	

Numerous microbial experiments have been employed in HE associated with chronic liver disease as these microbiota have demonstrated the ability to reduce blood ammonia levels and enhance mental function. However, there is currently limited research on microbial or FMT therapies regarding the progression of ALF to type A HE (Shen et al., 2015; Bloom et al., 2021; Häussinger et al., 2022). Further research is needed to determine whether microbiota intervention benefits animals or individuals with early ALF.
